# Evaluating the Effectiveness of Triiodothyronine Suppression and Withdrawal Versus Thyrogen Injections in Thyroid Cancer Assessments

**DOI:** 10.7759/cureus.51061

**Published:** 2023-12-25

**Authors:** Nada M Abdulhameed, Mazin A Janabi

**Affiliations:** 1 College of Medicine, Mohammed Bin Rashid University of Medicine and Health Sciences, Dubai, ARE; 2 Department of Nuclear Medicine, Mediclinic City Hospital, Dubai, ARE

**Keywords:** diagnostic assessment, thyroglobulin (tg), thyroidectomy, thyrogen recombinant tsh injections, thyroid cancer, triiodothyronine (t3) suppression

## Abstract

Objective

This study aimed to evaluate the specificity and effectiveness of triiodothyronine (T3) suppression and withdrawal, as compared to the conventional diagnostic approach using Thyrogen recombinant thyroid-stimulating hormone (TSH) injections, in the assessment of thyroid cancer patients post-thyroidectomy.

Methods

In this retrospective study, 18 patients diagnosed with thyroid cancer at a tertiary care hospital (Mediclinic City Hospital) in Dubai were included. The patients underwent total thyroidectomy, iodine ablation, and neck ultrasound. The cohort's clinical characteristics were analyzed, and histopathological examination of thyroid nodules was performed. In this study, paired T-tests were applied to evaluate the before-and-after impact of T3 and Thyrogen treatments on TSH and thyroglobulin (TG) levels in individual patients. To further analyze the effectiveness of these treatments, independent T-tests were conducted, allowing for a comparison of TSH and TG levels between different treatment groups within the patient cohort. This approach provided a comprehensive assessment of the treatments' effects on key thyroid indicators. Additionally, the diagnostic accuracy of T3 withdrawal and Thyrogen post-test on TG levels was assessed using statistical measures including sensitivity, specificity, and predictive values.

Results

The cohort had a mean age of 42.1 years and a female predominance. Distinct clinical profiles were observed across different thyroid cancer subtypes. Histopathological analysis confirmed typical features of papillary carcinoma variants. Significant changes in TSH levels post-treatment were noted, with T3 treatments showing a marked increase in TSH and TG levels, although changes in TG levels were not always statistically significant. Diagnostic test evaluation showed a sensitivity of 77.78%, a specificity of 83.33%, and an overall accuracy of 80.00% for T3 withdrawal and Thyrogen post-test on TG.

Conclusion

The study provides comprehensive insights into the clinical profiles and treatment responses in thyroid cancer patients post-thyroidectomy. The effectiveness of T3 and Thyrogen treatments in altering TSH and TG levels was established, with significant implications for patient management. The diagnostic tests for T3 withdrawal and Thyrogen post-test on TG demonstrated high accuracy, underlining their clinical utility in the post-treatment evaluation of thyroid cancer patients.

## Introduction

Thyroid carcinoma, the most common endocrine neoplasm and the 12th most prevalent cancer type, has witnessed a near doubling in incidence since 2000, now representing 2.1% of all cancer diagnoses [1,2]. The median five-year survival rate for thyroid cancer from 2009 to 2015 was a promising 98%. However, survival rates vary considerably among different subtypes. Anaplastic thyroid cancer (ATC), though rare and representing less than 1% of thyroid cancers, has a notably poor prognosis. Patients with ATC typically have a median survival of only three to seven months and a one-year survival rate between 10 and 20% [2,3]. In studies from the USA conducted in 2019, the mean age for thyroid cancer diagnosis is reported to be 48±16 years, and females represent 75% of the cases [4]. Contrastingly, in Middle Eastern patients, the median age of diagnosis is substantially lower, at 39.2 years, with an age range spanning from 18 to 88 years [5]. The geriatric population, though smaller in number, faces a more challenging prognosis due to heightened treatment-related morbidity and mortality. It has been found that older adults with differentiated thyroid cancer (DTC) are less likely to undergo surgery or receive radioactive iodine treatment compared to younger adults. This reduced treatment intensity in older patients (aged 65 and over) is associated with worse disease-specific survival [6]. In the post-thyroidectomy setting, Thyrogen injections have become integral in detecting residual thyroid tissue and recurrence, augmented by Thyrogen-stimulated TG testing [7].

In the conventional approach, patients undergo total thyroidectomy followed by radioiodine ablation and are subsequently treated with thyroxine (T4) to suppress thyroid-stimulating hormone (TSH) production [8], minimizing the risk of TSH-induced tumor growth. However, for assessing the recurrence of thyroid cancer, periodic withdrawal of thyroid hormone therapy (THT) is employed to elevate endogenous TSH levels, enhancing the sensitivity of radioiodine scanning and thyroglobulin (TG) testing [9]. In this context, Thyrogen injections, which contain a recombinant form of human TSH, have revolutionized the diagnostic process [10]. While they eliminate the need for THT withdrawal, potentially reducing sensitivity in detecting residual or recurrent cancer, they also raise concerns about cost and accessibility [11]. These injections are notably more expensive than traditional THT withdrawal, potentially limiting their widespread use, especially in resource-constrained settings. Additionally, while generally well-tolerated, some patients may experience mild adverse effects such as headaches and nausea, impacting patient comfort and compliance [12]. Moreover, the use of Thyrogen does not negate the need for continuous, lifelong monitoring of thyroid cancer, imposing a persistent burden on patients. Therefore, given the significant 20% recurrence rate in thyroid cancer, the quest for more accurate diagnostic methods to differentiate recurrence from treatment-related changes is crucial [4,13].

T3 suppression and withdrawal is an alternative diagnostic approach in the assessment of thyroid cancer, particularly after thyroidectomy [14,15]. This method involves initially suppressing the production of TSH by administering triiodothyronine (T3), and then temporarily ceasing its administration. The withdrawal leads to a natural spike in the body's TSH levels, which is crucial for diagnostic purposes. This increase in TSH is beneficial for enhancing the uptake of radioactive iodine during scanning procedures and for increasing the sensitivity of TG testing, both of which are key in detecting residual or recurrent thyroid cancer [15,16].

The potential benefits of T3 suppression and withdrawal lie in its ability to provide a more accurate assessment of thyroid cancer. Unlike Thyrogen injections, which artificially elevate TSH levels, T3 withdrawal allows for a natural increase in TSH [17], which may more effectively stimulate residual thyroid tissue or cancer cells, thereby improving the detection of any remaining disease. This method can be particularly valuable in patients where Thyrogen injections are not effective or feasible. Furthermore, T3 suppression and withdrawal can be a more cost-effective approach compared to the more expensive Thyrogen injections, making it an accessible option in various healthcare settings. Thus, T3 suppression and withdrawal presents itself as a viable alternative, potentially improving the accuracy and cost-effectiveness of thyroid cancer assessment [18].

Thyrogen, the current standard for stimulating thyroid tissue and facilitating cancer detection, may not always represent the most effective approach. T3 suppression and withdrawal, a more traditional method, might offer a heightened level of diagnostic accuracy. Improved detection of residual or recurrent thyroid cancer could lead to more tailored treatments, possibly reducing recurrence rates and improving overall patient prognosis. Therefore, the comparative analysis is essential to potentially improve diagnostic accuracy and patient outcomes in a field where precision is paramount.

This groundbreaking study in the United Arab Emirates (UAE) evaluates two thyroid cancer diagnostic methods, setting a precedent in the region and offering vital insights into this endocrine malignancy. Its potential to revolutionize UAE's clinical practices could lead to enhanced patient outcomes and influence future research and national guidelines, impacting thyroid cancer care both regionally and globally.

This article expands upon research originally presented as a meeting abstract at the 4th Annual Mediclinic Middle East Research Conference on August 20, 2021.

## Materials and methods

Ethical approval

This study received necessary ethical approvals from two committees, ensuring adherence to established ethical guidelines. The MBRU Institutional Review Board (MBRU-IRB) granted the first approval (reference number SRP-2018-036). The second approval came from the Mediclinic Research Committee (reference number MCME.CR.SR.139.MCIT.2020).

Study design

This study adopts a retrospective analytical cohort design, focusing on an in-depth review and analysis of medical records from 18 thyroid cancer patients. These patients underwent both T3 suppression and withdrawal, as well as thyrogen-stimulated protocols at Mediclinic City Hospital (MCH). The records, spanning from January 1, 2019, to September 2020, provided a comprehensive basis for comparing these diagnostic approaches in thyroid cancer management.

Selection criteria and enrollment of participants

The study commenced with the retrieval of a patient roster from the Information Technology (IT) department and the nuclear medicine clinic at MCH. A thorough selection process was undertaken to identify patients suitable for the study based on specific inclusion and exclusion criteria.

For inclusion, the study targeted patients who had a documented history of total thyroidectomy and those who had undergone iodine ablation treatment. Additionally, it included patients who had received both T3 suppression and withdrawal treatments, as well as Thyrogen injections. These criteria were essential to ensure that the study participants had a relevant medical history and treatment experience pertinent to the research objectives.

In contrast, the exclusion criteria were defined to omit certain patient groups from the study to maintain its focus. Patients who had undergone partial or other types of thyroidectomy, which did not fully align with total thyroidectomy, were excluded. This distinction was crucial to ensure the homogeneity of the surgical experiences of the participants. Furthermore, patients without a history of iodine ablation were also excluded, as the absence of this treatment could potentially affect the outcomes the study aimed to investigate.

This selection process led to the identification of 18 eligible patients who had undergone total thyroidectomy and iodine ablation and were also treated with both T3 suppression and withdrawal, along with Thyrogen injections, ensuring a consistent and relevant patient profile for the study and were currently receiving care at MCH. 

Data sources and collection methods

Acquisition of Medical Record Numbers (MRNs)

The initial phase of data sourcing involved obtaining the MRNs of eligible participants from the IT department at MCH. This process was governed by the criterion of selecting patients diagnosed with thyroid cancer who had undergone total thyroidectomy and iodine ablation.

Data Documentation and Training

To ensure the accuracy and reliability of data retrieval, a clinical researcher tasked with extracting information from the Hospital Information System (HIS) underwent a rigorous two-hour training program. This training was aimed at equipping the researcher with the necessary skills for proficient HIS utilization. The data entry process was closely overseen by either a clinician or their nursing staff to verify the accuracy and completeness of the data.

Data Collection and Analysis

Data collection was conducted from September to November 2020 at MCH, a premier tertiary healthcare institution in Dubai, UAE. The medical records procured from MCH constituted the primary data source for this study. Subsequent to data collection, a detailed analysis was performed at Mohammed Bin Rashid University (MBRU), specifically within the College of Medicine located in Dubai Health Care City. This analytical phase was crucial for a comprehensive and region-specific examination of the collected data, ensuring its relevance and applicability to the local healthcare context.

Diagnostic protocols in focus

T3 Suppression Protocol

This involved the temporary cessation of T3 thyroid hormone medication to induce a state of reduced T3 levels in the body. The body's response and functioning during this period were then assessed, aligning with established practices in thyroid cancer management [19].

Recombinant TSH (Thyrogen) Injection Protocol

This protocol entailed the administration of synthetic TSH to stimulate the thyroid gland. Widely used in the post-thyroidectomy management of thyroid cancer, this approach facilitates diagnostic testing and follow-up assessments, leveraging the capabilities of synthetic TSH [18,19].

Study variables and data collection

Sociodemographic and Clinical Variables

The study meticulously gathered sociodemographic data, including age, gender, nationality, and the presence of any comorbid conditions. The range of comorbidities considered for each participant extended to diabetes, hypertension, hyperlipidemia, other cancer diagnoses, cardiovascular diseases, and Hashimoto's thyroiditis. This comprehensive approach ensured a holistic understanding of each patient's health profile.

Clinical Investigations and Cancer Typing

Detailed clinical assessments were conducted, which included neck ultrasounds, identification of the specific type of thyroid cancer, evaluation of lymph node involvement, assessment of distant metastasis, and thorough histopathological examinations. The focus was narrowed down to two principal types of thyroid cancer for this study: papillary thyroid carcinoma and follicular thyroid carcinoma.

Laboratory Measurements and Testing Protocols

Key laboratory data were collected, specifically focusing on TSH and TG levels. These measurements were taken twice for each participant: once following the T3 suppression/withdrawal and again after the administration of Thyrogen injections, with a three-month interval between these two testing periods. It is crucial to note that the sequence of interventions involved T3 suppression and withdrawal being implemented prior to the Thyrogen injections, a factor significant in the interpretation of the results.

Statistical analysis

For the detailed assessment of the treatment effects on thyroid function markers, statistical analyses were conducted using both descriptive and inferential statistics. Descriptive statistics outlined patient characteristics and included mean±SD for continuous variables such as age and frequencies for categorical variables. Inferential statistics involved paired and independent sample t-tests to compare TSH and TG levels across different thyroid treatments, with assumptions for the t-tests thoroughly checked and met. The level of statistical significance was set at α ≤ 0.05. All analyses were done using IBM SPSS Statistics for Windows, Version 20 (Released 2011; IBM Corp., Armonk, New York, United States). In the assessment of diagnostic metrics, the test data was systematically classified into 'normal' and 'abnormal' categories based on established normal ranges for key biomarkers. Specifically, for TSH, a value greater than 30 uIU/mL was considered within the normal range. Similarly, for TG, a level below 0.04 ng/mL was deemed to be in the normal range.

## Results

Participants

This study encompassed 18 patients diagnosed with thyroid cancer. The mean age of the cohort was 42.1 years, ranging from 31 to 58 years old. All participants, without exception, underwent neck ultrasound, total thyroidectomy, and iodine ablation as part of their treatment. A notable majority of the patients were female, constituting between 60% and 100% of various thyroid cancer subtypes. Males represented 33.3% of the overall study group, with their prevalence varying across different cancer types. Specifically, the follicular thyroid carcinoma subgroup had no male patients.

Descriptive data

Table 1 shows the comprehensive characteristics and outcomes of thyroid cancer patients post-total thyroidectomy and iodine ablation. In the case of papillary thyroid carcinoma (N=10), the average age was 43.4 years. The gender distribution was 60% female and 40% male. All patients in this group underwent total thyroidectomy, iodine ablation, and neck ultrasound. Lymph node involvement was recorded in 16.7% of these cases (average: 9.3 lymph nodes), and there were no instances of distant metastasis. Comorbidities included hypertension in one patient and hypercholesteremia in another. Additionally, one patient (10%) had a history of Hashimoto's thyroiditis, with no other cancer types reported. For multifocal papillary thyroid carcinoma (N=6), the mean age was 39.0 years, with a female prevalence of 66.7% and a male prevalence of 33.3%. Similar to the previous group, all patients received total thyroidectomy, iodine ablation, and neck ultrasound. Around 33.3% had lymph node involvement (average: 6.5 lymph nodes), and 16.7% exhibited distant metastasis. As for comorbidities, one patient had hypertension, and another had hypercholesteremia. There were no cases of Hashimoto's thyroiditis or other cancers in this group. In the follicular thyroid carcinoma subgroup (N=2), the mean age was 45 years, with all patients being female. These patients also underwent the full treatment regimen of total thyroidectomy, iodine ablation, and neck ultrasound. There were no instances of lymph node involvement, distant metastasis, hypertension, hypercholesteremia, or other cancers in this group. Notably, one patient (50%) had Hashimoto's thyroiditis. 

**Table 1:** Characteristics and Outcomes of Thyroid Cancer Patients Post-total Thyroidectomy and Iodine Ablation at MCH, a Tertiary Care Hospital in Dubai, United Arab Emirates, 2020 Data presented in brackets represent the percentage of the sample. HTN: Hypertension

Characteristics and clinical outcomes	Overall (N=18)	Papillary Thyroid Carcinoma (N=10)	Multifocal Papillary Thyroid Carcinoma (N=6)	Follicular Thyroid Carcinoma (N=2)
Age Mean (range)	42.1 (31-58)	43.4 (31-58)	39.0 (33-43)	45.0 (43-47)
Female	12 (66.7%)	6 (60%)	4 (66.7%)	2 (100%)
Male	6 (33.3%)	4 (40%)	2 (33.3%)	0
Total Thyroidectomy	18 (100%)	10 (100%)	6 (100%)	2 (100%)
Iodine Ablation	18 (100%)	10 (100%)	6 (100%)	2 (100%)
Neck Ultrasound	18 (100%)	10 (100%)	6 (100%)	2 (100%)
Number of Patients with lymph node involvement	5 (27.8%)	3 (16.7%)	2 (33.3%)	0
Number of Lymph nodes involved Mean (range)	8.2 (2-19)	9.3 (3-19)	6.5 (2-11)	0
Distant Metastasis	1 (5.6%)	0	1 (16.7%)	0
Diabetes	0	0	0	0
HTN	1	0	1 (16.7%)	0
Hypercholesteremia	1	0	1 (16.7%)	0
Hashimoto’s Thyroiditis	2 (11.1%)	1 (10%)	0	1 (50%)
Other cancers	0	0	0	0

Outcome data

The histopathological analysis of the thyroid nodules revealed significant insights, echoing the study's results. Key histological features, such as nuclear grooves, pseudo-inclusions, papillary structures, and occurrences of Hashimoto's thyroiditis, were consistently aligned with the defining characteristics of papillary carcinoma variants, including their patterns of infiltration and related attributes. This congruence between observed histological features and the descriptions provided by the results underscores the reliability and precision of the diagnostic methods employed, primarily based on histopathological evaluations (Table 2).

**Table 2:** Histopathological Profiles of Thyroid Nodules in the Study Cohort

Patient	Pathophysiology
1	Papillary carcinoma, conventional variant, with features of dense, occasionally hemorrhagic nodules, mixed follicular cell populations, and various structural arrangements. Occasional grooves, pseudo-inclusions, and the presence of colloid material and foamy histiocytes in a blood-stained background are noted, with no calcifications.
2	Soft tissue infiltration by papillary carcinoma cells, lymph node involvement with metastatic carcinoma and extra-capsular spread, neoplastic masses with enlarged nuclei displaying ground glass appearance, nuclear grooves, membrane irregularities, pseudo-inclusions, and Hurthle cell change.
3	A cystic mass with papillary frond-like structures, characterized by a thin-walled cyst lined by bland flattened epithelium. The area of papillary growth shows complex architecture with atypical epithelial cells displaying thyroid papillary carcinoma features and psammomatous microcalcifications.
4	Nuclear grooves, membrane irregularity, and pseudo-inclusions, suggestive of well-differentiated papillary carcinoma.
5	Focal lymphocytic thyroiditis, multi-nodular hyperplasia in the right lobe, and multiple papillary carcinoma foci with mixed architecture. Neoplastic masses have enlarged, crowded nuclei with characteristic features and are accompanied by lymphocytic Hashimoto's thyroiditis.
6	Predominantly uniform cells with microfollicular structure, lacking colloid, suggesting a follicular neoplasm.
7	Intensive lymphocyte and plasma cell infiltration, along with reactive lymphoid follicles, causing loss of thyroid follicles and localized Hurthle cell changes. A small lesion exhibits papillary and follicular features, likely indicating well-differentiated papillary carcinoma in a background of Hashimoto's thyroiditis.
8	Follicular structure. White nodule measuring 3mm corresponds to papillary proliferation of which the nuclei appear irregular and are piled up. multi-incised, and overlapping..
9	Follicular structure. White nodule measuring 3mm corresponds to papillary proliferation of which the nuclei appear irregular and are piled up. multi-incised, and overlapping..
10	An isthmus thyroid nodule with mildly to moderately cellular smears containing uniform follicular cells in monolayered sheets and small clusters. The background contains colloid and hemorrhage, with no signs of malignancy like nuclear grooving or pseudo inclusions.
11	A single group of atypical cells with calcified material within the cell block.
12	Multifocal papillary thyroid carcinoma in multiple foci involving both lobes and the isthmus. The tumor is moderately differentiated with capsular and perineural invasion, along with nuclear grooves and intra-nuclear inclusions.
13	Papillary carcinoma combined with follicular architecture, displaying multiple papillae lined by cells with nuclear grooves and intra-nuclear inclusions, along with an area of calcification.
14	Classical papillary carcinoma of the thyroid composed of multiple papillae lined by cells with nuclear grooves and intra-nuclear inclusions.
15	Classical papillary carcinoma of the thyroid composed of multiple papillae lined by cells with nuclear grooves and intra-nuclear inclusions.
16	Capsular invasion, with crowded uniform cells arranged in a microfollicular architecture.
17	Right lobe thyroid nodule exhibiting classical papillary thyroid carcinoma with nuclear grooves, intra-nuclear inclusions, calcifications (Psammoma bodies), and vascular invasion.
18	Non-encapsulated micropapillary neoplasm, combining papillary microcarcinoma with follicular architecture. Mild pleomorphism is identified.

Impact of T3 and Thyrogen treatments on TSH and TG levels in Post-thyroidectomy patients

Table 3 shows the paired sample t-test analysis results, examining the impact of T3 and Thyrogen treatments on TSH and TG levels. Significant changes were observed in TSH levels, while TG levels exhibited more subtle variations. Under T3 treatment, TSH levels increased significantly from suppression (M = 0.122, SD = 0.170) to withdrawal ((M = 61.821, SD = 36.578), t(17) = 7.167, p < 0.001). TG levels also rose from suppression (M = 0.195, SD = 0.249) to withdrawal (M = 14.947, SD = 30.487), but this increase did not reach statistical significance, (t(14) = 1.882, p = 0.081). In the Thyrogen treatment group, a significant increase in TSH levels was observed from pre-treatment (M = 0.19, SD = 0.206) to post-treatment ((M = 109.687, SD = 22.788), t(14) = 18.672, p < 0.001). TG levels demonstrated a non-significant increase from pre-Thyrogen (M = 0.176, SD = 0.211) to post-Thyrogen ((M = 1.096, SD = 1.921), t(14) = 2.002, p = 0.065).

**Table 3:** Paired Sample T-test Analysis of TSH and TG Levels Following T3 and Thyrogen Treatments in Post-Thyroidectomy Patients TSH: Thyroid-stimulating hormone; TG: thyroglobulin; N: number of participants; Std. Deviation: standard deviation; df: degrees of freedom; *: Indicates a statistically significant difference at α=0.05

Treatment	Paired variables	N	Mean	Std. Deviation	95% Confidence Interval of the Difference		T statistics (df)=p
T3	Suppression TSH	18	0.122	0.170	43.537	79.861	7.167(17)=0.00*
	Withdrawal TSH	18	61.821	36.578	-	-	-
	Suppression TG	15	0.195	0.249	2.058	31.561	1.882(14)=0.081
	Withdrawal TG	15	14.947	30.487	-	-	-
Thyrogen	Pre TSH	15	0.19	0.206	96.920	122.075	18.672(14)=0.00*
	Post TSH	15	109.687	22.788	-	-	-
	Pre TG	15	0.176	0.211	0.065	1.905	2.002(14)=0.065
	Post TG	15	1.096	1.921	-	-	-

Comparative analysis of TSH and TG levels among different thyroid treatments in patients

Table 4 presents the results of the independent sample t-test analysis, comparing TSH and TG levels across different treatments in a sample of patients. The results indicate that for TSH levels, there was no significant difference between the T3 suppression treatment (M = 0.137, SD = 0.175) and pre-Thyrogen treatment ((M = 0.200, SD = 0.210), p = 0.378). Similarly, TG levels did not significantly differ between the T3 suppression treatment (M = 0.201, SD = 0.245) and pre-Thyrogen treatment ((M = 0.192, SD = 0.210), p = 0.921). This suggests that neither TSH nor TG levels were significantly altered by these treatments when compared to each other. In contrast, a significant change was observed in TSH levels when comparing the T3 withdrawal treatment (M = 74.052, SD = 25.180) to post-Thyrogen treatment ((M = 110.093, SD = 23.602, p = 0.00). This indicates a statistically significant increase in TSH levels after Thyrogen treatment compared to T3 withdrawal. However, for TG levels, while the mean increased from the T3 withdrawal treatment (M = 14.951, SD = 30.485) to post-Thyrogen treatment (M = 1.177, SD = 1.967), this change was not statistically significant (p = 0.103).

**Table 4:** Independent Sample T-test Analysis of TSH and TG Levels Between T3 Suppression, Pre-Thyrogen, T3 Withdrawal, and Post-Thyrogen Treatments in Patients TSH: Thyroid-stimulating hormone; TG: thyroglobulin; T3: triiodothyronine; df: degrees of freedom; *: indicates a statistically significant difference at α=0.05

Marker	Treatment	N	Mean	Std. Deviation	df	p value
TSH	T3 Suppression	16	0.137	0.175	28	0.378
	Pre Thyrogen	14	0.200	0.210	-	-
	T3 withdrawal	15	74.052	25.180	27	0.000*
	Post Thyrogen	14	110.093	23.602	-	-
TG	T3 Suppression	15	0.201	0.245	27	0.921
	Pre Thyrogen	14	0.192	0.210	-	-
	T3 withdrawal	15	14.951	30.485	27	0.103
	Post Thyrogen	14	1.177	1.967	-	-

Performance metrics of the medical diagnostic test

Tables 5, 6 present a detailed evaluation of the accuracy and effectiveness of two diagnostic tests - T3 Withdrawal and Thyrogen post-test in measuring TG levels. It includes essential statistical measures such as sensitivity and specificity, which indicate the test's ability to correctly identify true positive and true negative results, respectively.

**Table 5:** Chi-Square Analysis of T3 Withdrawal on TG and Thyrogen Post-test on TG Results in Patients *: Indicates statistically significant difference at α=0.05; TG: thyroglobulin

Treatments groups	Thyrogen Post-test ranged TG	Thyrogen Post-test ranged TG	Total	Chi-square	P value
T3 Withdrawal Normal ranged TG	5 (83.30%)	1 (16.70%)	6 (100%)	5.402	0.02*
T3 Withdrawal Abnormal ranged TG	2 (22.20%)	7 (77.80%)	9 (100%)	-	-

**Table 6:** Evaluation of Diagnostic Test Accuracy for T3 Withdrawal on TG and Thyrogen Post-test on TG With Confidence Intervals *: These values are dependent on disease prevalence; TG: thyroglobulin

Statistic	Value	95% CI
Sensitivity	77.78%	39.99% to 97.19%
Specificity	83.33%	35.88% to 99.58%
Positive Likelihood Ratio	4.67	0.75 to 28.89
Negative Likelihood Ratio	0.27	0.07 to 0.95
Disease prevalence (*)	60.00%	32.29% to 83.66%
Positive Predictive Value (*)	87.50%	53.07% to 97.74%
Negative Predictive Value (*)	71.43%	41.16% to 89.93%
Accuracy (*)	80.00%	51.91% to 95.67%

## Discussion

This study represents the first of its kind in the UAE, focusing on the use of T3 suppression and withdrawal for assessing thyroid cancer patients. Our findings revealed that patients exhibited higher levels of TG following T3 suppression and withdrawal. In contrast, these same patients demonstrated lower TG levels after receiving Thyrogen recombinant TSH injections. This discrepancy suggests that low TG levels post-Thyrogen administration could potentially lead to a false impression of complete remission, underscoring the necessity for continued treatment. Consequently, our study indicates that T3 suppression and withdrawal offer greater sensitivity and specificity, enhancing the overall quality and outcomes of thyroid cancer assessments.

To date, no published studies have directly compared T3 suppression and withdrawal with Thyrogen recombinant TSH injections, particularly in the context of assessing thyroid cancer patients or in evaluating the diagnostic benefits of T3 suppression and withdrawal. Nevertheless, the efficacy of Thyrogen has been frequently documented. Duntas et al. investigated Thyrogen's role in both diagnosing and treating thyroid cancer, noting significant improvements in patient quality of life [20]. This was primarily attributed to the avoidance of hypothyroidism, a common side effect of traditional diagnostic and treatment methods. Their research highlighted that Thyrogen stimulation consistently increased serum TG levels across all patients, regardless of their basal serum TG levels, establishing Thyrogen's reliability and safety as a diagnostic tool and its potential in treating metastases.

Furthermore, Emerson et al. explored the pharmacological and clinical applications of recombinant human thyroid-stimulating hormone (rhTSH/Thyrogen), noting its specific activity of approximately 4 IU/mg and potent stimulatory effects on T4, T3, and TG secretion [21]. Their findings emphasize the utility of Thyrogen in preparing thyroidectomized thyroid cancer patients for whole-body iodide scans and serum TG measurements. Pellegriti et al. assessed the rhTSH/thyroglobulin test's accuracy in DTC patients, particularly those with persistent disease and low TG levels [22]. They suggested that optimizing TG measurement after rhTSH administration might require a dose adjustment and repeated blood sampling.

Conversely, Rodríguez-Molinero et al. presented a case study highlighting the benefits of antithyroid drugs and exogenous T3 administration in patients with metastatic cancers [23]. Their findings indicate the potential of plasma T3 levels as a novel marker for progression in certain types of cancer, offering insights that align with our study. Additionally, Tosovic et al. examined the correlation between serum T3 levels and breast cancer aggressiveness [24]. Their study found a positive association between higher prediagnostic T3 levels and increased tumor size, lymph node metastases, and negative estrogen and progesterone receptor status, highlighting the intricate interplay between thyroid hormones and cancer progression. While Thyrogen is recognized as a dependable diagnostic and therapeutic agent, particularly in preventing hypothyroidism-related complications, the role of T3 is more complex, influencing tumor progression and treatment effectiveness. Our study adds to this dialogue, offering fresh insights into the comparative efficacy of these treatments in a specific group of thyroid cancer patients.

The findings from our study are poised to enhance the quality of assessments, facilitate earlier detection of cancer recurrence, and reduce the rate of false negatives. This will guide the treatment of thyroid cancer patients, particularly those who have undergone total thyroidectomy. Furthermore, our research could inform the development of new national guidelines. To our knowledge, this study is the first of its kind in the UAE, comparing the use of T3 suppression and withdrawal to Thyrogen recombinant TSH injections in terms of quality of assessment, sensitivity, specificity, and guiding the treatment course. A main advantage is that multiple outcomes were able to be assessed. There are no ethical considerations since the review of the medical records was blind and every patient had a unique file identification number, and the data was anonymized.

However, a significant limitation of this research is the reliance on a very small sample size, which inherently restricts the breadth and depth of the conclusions that can be drawn. This limitation is further compounded by the fact that the research population was confined to a single hospital in the Emirate of Dubai, MCH. As MCH is part of the private sector, its patient demographic may not accurately represent the broader UAE population, thereby limiting the external validity of our findings and potentially introducing selection bias. Moreover, the patients included in the study were selected through a process of random sampling and come from varied pathological backgrounds, which, while beneficial for diversity, further emphasizes the challenges posed by the small sample size in making broad generalizations. As mentioned, this study was limited with respect to the data provided and with regard to obtaining medical records from a single hospital, in only one Emirate of the UAE, Dubai. In view of this limitation, future research may need to consider allocating different hospitals from all around the UAE, including a wide spectrum of governmental and private hospitals.

## Conclusions

This study showed that T3 suppression and withdrawal are more natural, beneficial, specific, and sensitive in terms of quality of assessment when compared to the current standardized test, Thyrogen-recombinant TSH injections. TG levels were much higher when patients underwent T3 suppression and withdrawal, indicating the need for further treatment. The low levels of TG after Thyrogen recombinant TSH injection were misleading as false negatives. T3 suppression and withdrawal are therefore recommended to be used instead of Thyrogen recombinant TSH injections. These findings may help inform physicians about the importance of T3 suppression and withdrawal and help in establishing a national guideline.
